# Illustrating the structures of bias from immortal time using directed acyclic graphs

**DOI:** 10.1093/ije/dyae176

**Published:** 2025-01-07

**Authors:** Guoyi Yang, Stephen Burgess, C Mary Schooling

**Affiliations:** 1 School of Public Health, Li Ka Shing Faculty of Medicine, The University of Hong Kong, Hong Kong, China; 2 MRC Biostatistics Unit, University of Cambridge, Cambridge, UK; 3 British Heart Foundation Cardiovascular Epidemiology Unit, Department of Public Health and Primary Care, University of Cambridge, Cambridge, UK; 4 Graduate School of Public Health and Health Policy, City University of New York, New York, United States

**Keywords:** bias, directed acyclic graphs, epidemiology, immortal time

## Abstract

**Background:**

Immortal time is a period of follow-up during which death or the study outcome cannot occur by design. Bias from immortal time has been increasingly recognized in epidemiological studies. However, the fundamental causes and structures of bias from immortal time have not been explained systematically.

**Methods:**

We use an example “Does winning a Nobel Prize prolong lifespan?” for illustration. We illustrate how immortal time arises and present structures of bias from immortal time using directed acyclic graphs that specify time-varying variables. We further explore the structures of bias with the exclusion of immortal time and with the presence of competing risks. We discuss how these structures are shared by different study designs in pharmacoepidemiology and provide solutions, where possible, to address the bias.

**Results:**

The fundamental cause of immortal time is misalignment of exposure allocation and eligibility. Specifically, immortal time arises from using post-eligibility information to define exposure or using post-exposure information to define eligibility. The structures of bias from immortal time are confounding by survival until exposure allocation or selection bias from selecting on survival until eligibility. Excluding immortal time from follow-up does not fully address this confounding or selection bias, and the presence of competing risks can worsen the bias. Bias from immortal time may be avoided by aligning baseline, exposure allocation and eligibility, and by excluding individuals with prior exposure.

**Conclusions:**

Understanding bias from immortal time in terms of confounding or selection bias helps researchers identify and thereby avoid or ameliorate this bias.

## Introduction

Immortal time refers to a period of follow-up during which death or the study outcome cannot occur by design.^1^ Bias from immortal time was first identified in the 1970s,^2, 3^ and has been described as a bias resulting from counting follow-up times incorrectly in terms of exposure status.^4, 5^ This bias has been named “immortal time bias”,^4, 6, 7^ “survivor treatment selection bias”,^8^ “survivor bias”,^9^ or generally “time-dependent bias” or “time-related bias”.^10–12^ Although bias from immortal time has been warned against for decades, it is still increasingly evident in epidemiological studies.^6, 7, 10–14^ This is possibly because the fundamental causes and structures of bias from immortal time have not been explained systematically using a structural approach.

Directed acyclic graphs (DAGs) are useful in illustrating causal structures and thereby preventing the key sources of bias in epidemiological studies, i.e., confounding (the existence of common causes of exposure and outcome) and selection bias (conditioning on common consequences of exposure and outcome).^15–17^ Previous DAGs have suggested that the structure of bias from immortal time is misclassification,^18, 19^ selection bias,^18, 19^ or collider stratification bias.^20^ Building on these previous works, we illustrate that the fundamental cause of immortal time is misalignment of exposure allocation and eligibility. Specifically, immortal time can arise through two mechanisms, that is using post-eligibility information to define exposure and using post-exposure information to define eligibility. We present the structures of bias from the first type of immortal time as confounding (classical immortal time bias) and bias from the second type of immortal time as selection bias using DAGs that specify time-varying variables.^21, 22^ We further explore the structures of bias with the exclusion of immortal time and with the presence of competing risks. We discuss how these structures are shared by different study designs in pharmacoepidemiology^4, 6^ and provide solutions, where possible, to address the bias.

### Does winning a Nobel Prize prolong lifespan?

We use an example “Does wining a Nobel Prize prolong lifespan?” for illustration, because of the time lag between publication of a scientific discovery and conferment of a Nobel Prize.^23^ Consider a study to investigate the survival benefit of winning a Nobel Prize. All scientists who won at least one Nobel Prize were identified as Nobel Prize winners. For each winner, a control was selected as a scientist who was the same sex, was born in the same era, worked in the same institution, and published a similar work when the winner’s discovery was published. For simplicity, we suppose there are no other confounders nor delayed effects of winning a Nobel Prize and other causes of death on lifespan.

To specify time-varying variables, we use DAGs including separate nodes for a variable at different times.^21, 22^ The full DAG from publication of the discovery (time 0) to the end of follow-up (time k+) is provided in Supplementary Figure S1. For illustration, we suppose k=2 and use two time points (1 and 2). E_1_ and E_2_ denote exposure status at time 1 and 2, respectively. D_1+_ and D_2+_ denote outcome status between time 1 and 2 and after time 2, respectively. U_1_ and U_2_ denote status of another unmeasured cause of the outcome at time 1 and 2, respectively. E_2_ is the exposure of interest (winning at least one Nobel Prize at or before time 2), D_2+_ is the outcome of interest (death at time 2+), and the arrow from E_2_ to D_2+_ is the causal effect of interest. In a time-to-event analysis, the exposure variable is E_2_ and the outcome variable is (D_2+_, T), where T is the time variable. T starts from baseline and ends until time 2+ or until occurrence of the outcome, whichever occurs earlier.

#### Immortal time arises from using post-eligibility information to define exposure

**Figure 1:**
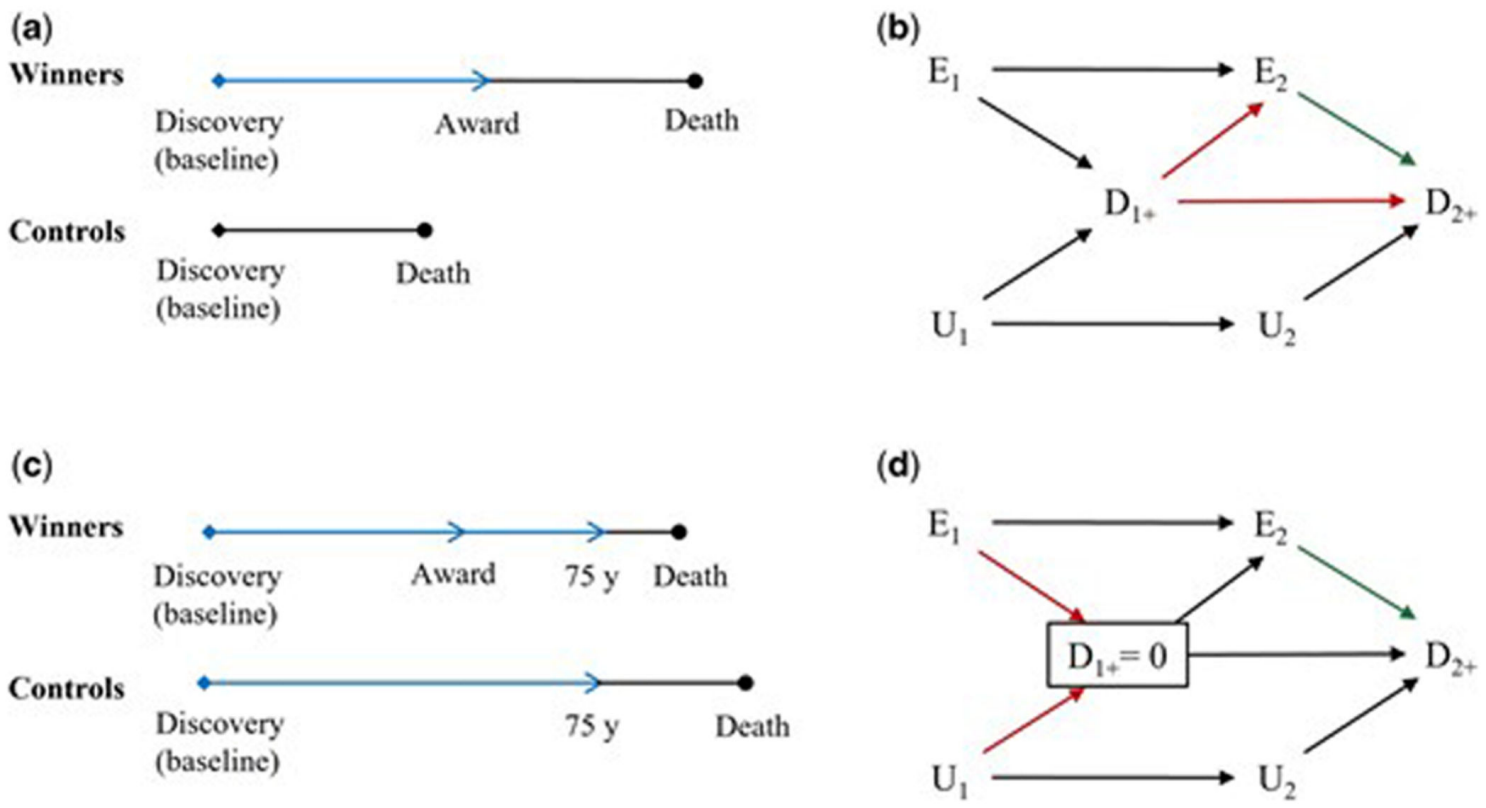
Illustrations and directed acyclic graphs of study designs with immortal time. Blue line denotes immortal time. E_1_ and E_2_ are exposure status at time 1 and 2, respectively. D_1+_ and D_2+_ are outcome status between time 1 and 2 and after time 2, respectively. U_1_ and U_2_ are status of another unmeasured cause of the outcome at time 1 and 2, respectively. Red arrows denote key arrows that create open paths and result in bias; green arrow denotes the causal effect of interest. (a) Baseline was set as the day when the discovery was published; (b) Immortal time arises from using post-eligibility information to define exposure; (c) Baseline was set as the day when the discovery was published and all scientists who died before age 75 years were excluded; (d) Immortal time arises from using post-exposure information to define eligibility.

Suppose baseline was set as the day when the discovery was published. Nobel Prize winners have to survive until they have won their first award to be classified as winners; however, there is no such requirement for controls. Immortal time refers to the time between publication of the discovery and conferment of the Nobel Prize for winners (Figure 1a), which arises from using post-eligibility information to define exposure.

The bias generated is depicted in Figure 1b. The arrow from E_1_ to E_2_ means scientists who won Nobel Prizes at time 1 were also Nobel Prize winners at time 2. The arrow from D_1+_ to D_2+_ means scientists who died between time 1 and 2 were also dead after time 2. The arrow from D_1+_ to E_2_ means the scientists who were not Nobel Prize winners at time 1 have to be alive from time 1 to 2 to be classified as Nobel Prize winners at time 2. The structure of this bias is confounding, that is the presence of a common cause (D_1+_) of the exposure (E_2_) and the outcome (D_2+_). It creates an open path between E_2_ and D_2+_, which can bias the association towards favouring the winners. Specifically, winners having to remain alive until the Nobel Prize is awarded means survival confounds the effect of winning the Nobel Prize on lifespan.

Classical immortal time bias arises from using post-eligibility information to define exposure. Suissa has reviewed cohort studies in pharmacoepidemiology leading to this bias.^4^ This bias also occurs in case-control studies, named “time-window bias”.^24, 25^ Specifically, these studies define exposure using the mean, minimum, or maximum number of treatments after recruitment (e.g., at least one treatment during follow-up).^4, 24^ Individuals have to be healthy enough to remain alive until they receive the treatment, which confounds the effect of treatment on health outcomes. As such, the fundamental issue is differences between the individuals who do and do not survive until the treatment (exposure allocation) rather than counting time incorrectly.

#### Immortal time arises from using post-exposure information to define eligibility

Suppose baseline was set as the day when the discovery was published. All scientists who died before age 75 years were excluded. Immortal time refers to the time between publication of the discovery and age 75 years for both winners and controls (Figure 1c), which arises from using post-exposure information to define eligibility.

The bias generated is depicted in Figure 1d. The box around D_1+_ means the analysis was restricted to scientists who remained alive until time 1+. The structure of this bias is selection bias, that is conditioning on a common consequence (D_1+_) of the exposure (E_1_) and another unmeasured cause of the outcome (U_1_). Although conditioning on D_1+_ closes the open backdoor path between E_2_ to D_2+_, it creates another open path between E_1_ and U_1_. If winning a Nobel Prize truly provides survival benefit, Nobel Prize winners who survive until 75 years are more likely to have another cause of death than controls who survive until 75 years without winning a Nobel Prize. Therefore, the association is biased towards the opposite direction of the true effect. The exception is when the exposure, winning a Nobel Prize, has no causal effect on the outcome, death; then D_1+_ is not a collider (Supplementary Figure S2). Specifically, selecting on survival to 75 years when previous Nobel Prize status and other factors affect survival creates the classic *M*-bias, here specifically butterfly bias^26^ given survival to 75 years affects current Nobel Prize status and subsequent survival (Figure 1d). The magnitude of *M*-bias is generally smaller than confounding bias.^26^ This *M*-bias can be ameliorated by adjusting for prior exposure E_1_ or a well measured U_1_.

In a pharmacoepidemiological study using post-exposure information to define eligibility (e.g., survival to one year after recruitment), selecting on survival to one year after recruitment when prior treatment and other factors affect survival also creates selection bias. Again, the fundamental issue is differences between the individuals who survive until eligibility with and without the treatment (exposure) rather than counting time incorrectly.

### Excluding immortal time from follow-up does not fully address the bias

Immortal time can be excluded from follow-up by redefining baseline to a later timepoint, for some or all individuals in the study. We further illustrate the structure of bias in study designs excluding immortal time from follow-up.

#### When immortal time arises from using post-eligibility information to define exposure

**Figure 2:**
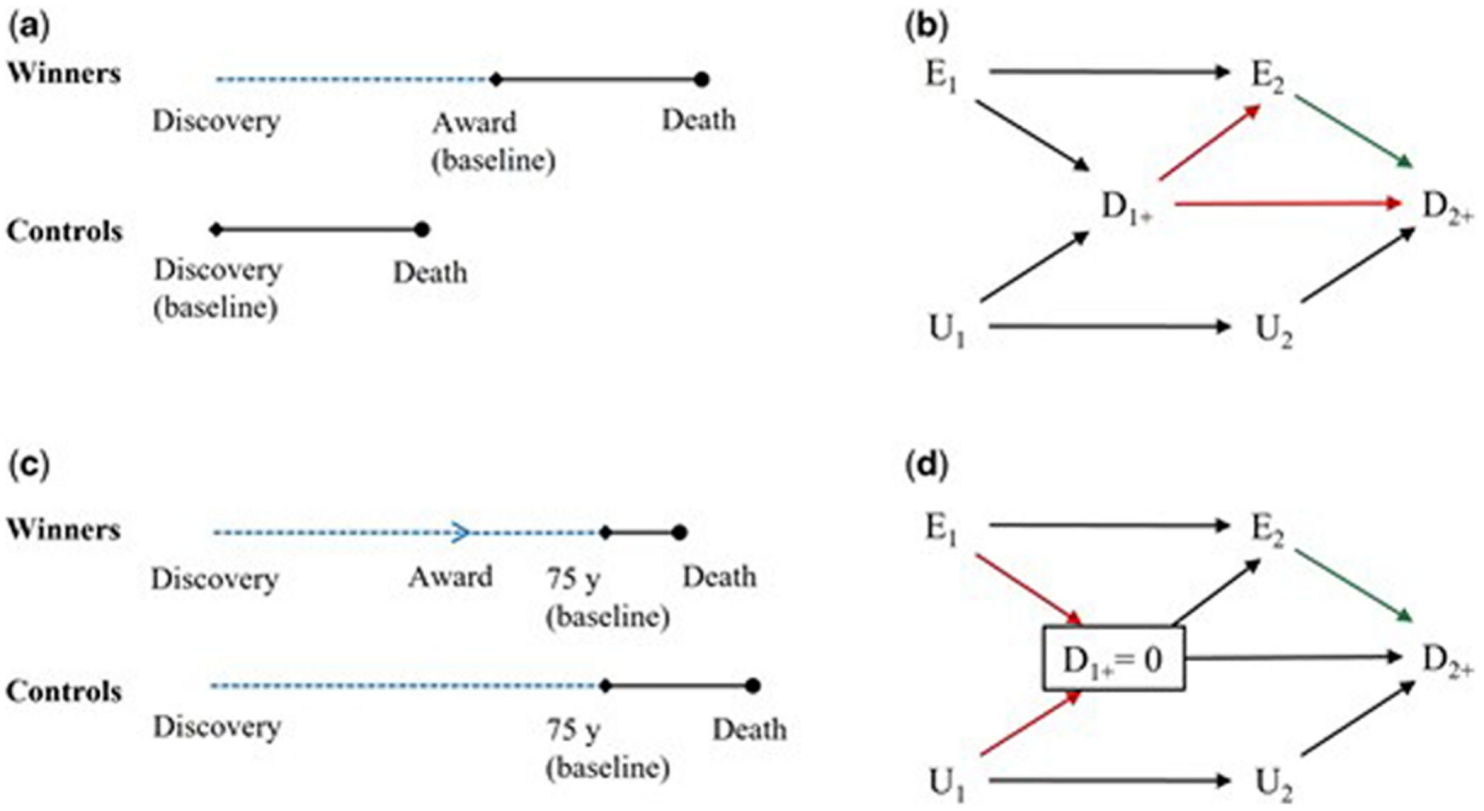
Illustrations and directed acyclic graphs of study designs excluding immortal time from the follow-up. Blue dotted line denotes immortal time excluded from the follow-up. E_1_ and E_2_ are exposure status at time 1 and 2, respectively. D_1+_ and D_2+_ are outcome status between time 1 and 2 and after time 2, respectively. U_1_ and U_2_ are status of another unmeasured cause of the outcome at time 1 and 2, respectively. Red arrows denote key arrows that create open paths and result in bias; green arrow denotes the causal effect of interest. (a) Baseline was set as the day when Nobel Prize winners won their first award for winners, but as the day when the discovery was published for controls; (b) Immortal time arising from using post-eligibility information to define exposure is excluded; (c) Baseline was set as age 75 years and all scientists who died before age 75 years were excluded; (d) Immortal time arising from using post-exposure information to define eligibility is excluded.

Suppose baseline was set as the day when Nobel Prize winners won their first award for winners, but as the day when the discovery was published for controls. Immortal time between publication of the discovery and the first award for winners is thereby excluded from follow-up (Figure 2a).

The DAG is depicted in Figure 2b. The arrow from D_1+_ to E_2_ persists because the scientists who were not Nobel Prize winners at time 1 still have to be alive from time 1 to 2 to be classified as Nobel Prize winners at time 2. Excluding immortal time from follow-up partially eliminates the guaranteed survival advantage of winners, but it does not close the open backdoor path between E_2_ and D_2+_. As such, the structure of this bias is confounding, which is the same as Figure 1b given DAGs do not capture quantitative information.^21, 22^

“Exposure-based” cohorts in pharmacoepidemiology use post-eligibility information to define exposure (e.g., at least one treatment during follow-up).^4^ Baseline is set as the time of the first treatment for the exposed group, but as the time of diagnosis for the unexposed group.^4^ Immortal time between diagnosis and the first treatment for the exposed group is thereby excluded from follow-up. However, classical immortal time bias persists, because the fundamental issue is differences between the individuals who do and do not survive until the treatment (exposure allocation) rather than counting time incorrectly.

#### When immortal time arises from using post-exposure information to define eligibility

Suppose baseline was set as age 75 years. All scientists who died before age 75 years were excluded. Immortal time between publication of the discovery and age 75 years for both winners and controls is excluded from follow-up (Figure 2c).

The DAG is depicted in Figure 2d. The box around D_1+_ means the analysis was restricted to scientists who remained alive until time 1+. Conditioning on D_1+_ creates an open path between E_1_ and U_1_. The exception is when the exposure, winning a Nobel Prize, has no causal effect on the outcome, death; then D_1+_ is not a collider (Supplementary Figure S2). Excluding immortal time from follow-up does not change the structure of bias. As such, the structure of this bias is selection bias, as in Figure 1d.

The landmark approach has been suggested to address classical immortal time bias in pharmacoepidemiology.^9, 27^ This approach sets a landmark time and classifies individuals as exposed and unexposed based on the treatment received before the landmark. The landmark is set as baseline and all individuals who die before the landmark are excluded. This approach has a similar structure as Figure 2d, except that it assesses the causal effect of E_1_ on D_2+_ (Supplementary Figure S3). Again, using post-exposure information to define eligibility reduces confounding (classical immortal time bias) at the expense of introducing selection bias, but it may provide unbiased results when the treatment has no effect on survival.^27^

### Competing risks can worsen bias from immortal time

A competing risk is an event which precludes occurrence of the outcome or alters the probability of occurrence of the outcome.^28^ Competing risks should be considered for outcomes other than all-cause mortality. Consider another study to investigate the effect of winning a Nobel Prize on the risk of dementia, where cardiovascular death is a competing risk. Nobel Prize winners and controls were identified as above.

In DAGs specifying time-varying variables, E_1_ and E_2_ are exposure status at time 1 and 2, respectively. D_1+_ and D_2+_ are outcome status between time 1 and 2 and after time 2, respectively. CR_1+_ and CR_2+_ are status of a competing risk between time 1 and 2 and after time 2, respectively. U_1_ and U_2_ are status of an unmeasured common cause of the outcome and the competing risk at time 1 and 2, respectively.

#### When immortal time arises from using post-eligibility information to define exposure

**Figure 3:**
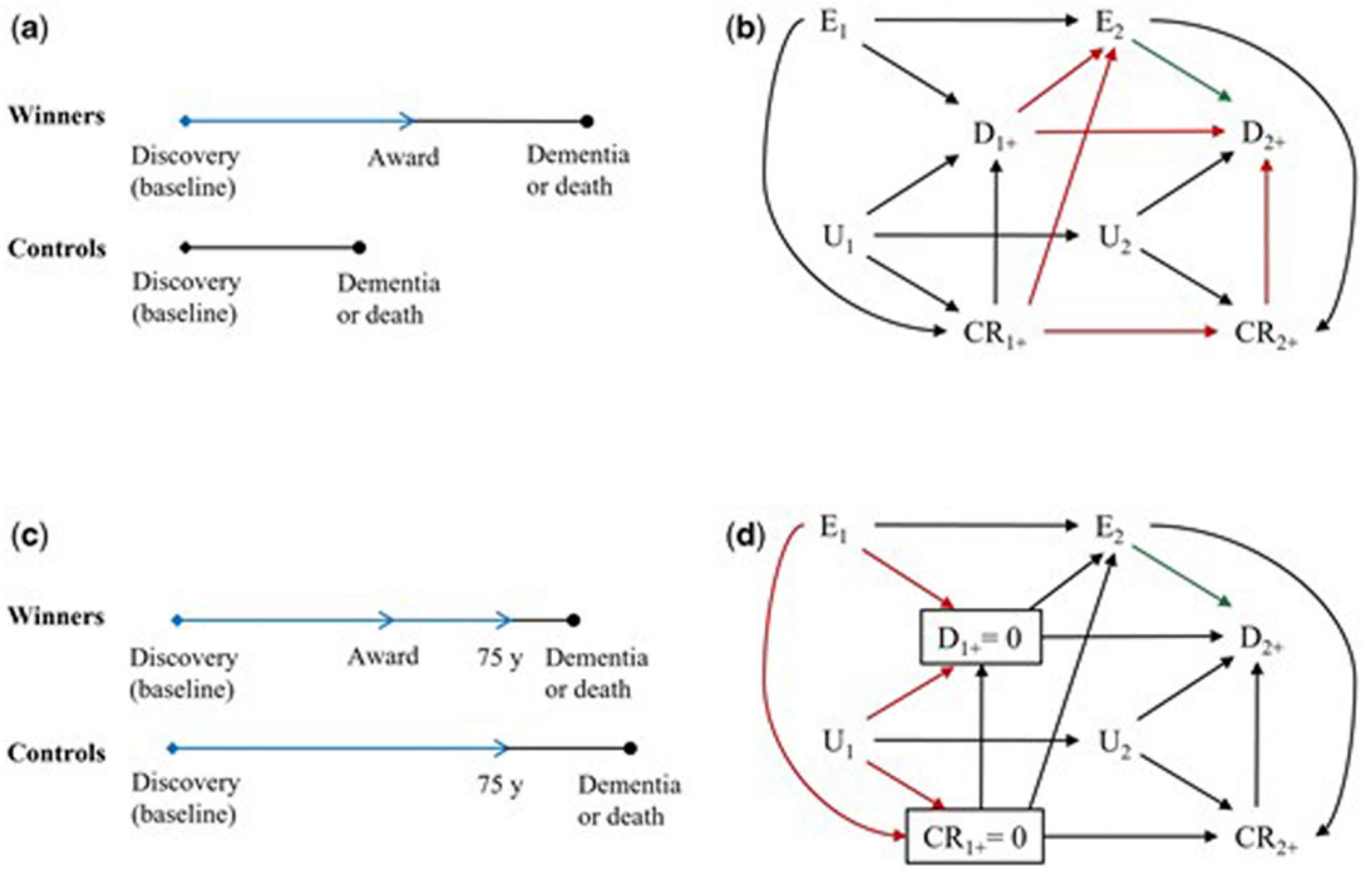
Illustrations and directed acyclic graphs of study designs with immortal time and the presence of a competing risk. Blue line denotes immortal time. E_1_ and E_2_ are exposure status at time 1 and 2, respectively. D_1+_ and D_2+_ are outcome status between time 1 and 2 and after time 2, respectively. CR_1+_ and CR_2+_ are status of a competing risk between time 1 and 2 and after time 2, respectively. U_1_ and U_2_ are status of an unmeasured common cause of the outcome and the competing risk at time 1 and 2, respectively. Red arrows denote key arrows that create open paths and result in bias; green arrow denotes the causal effect of interest. (a) Baseline was set as the day when the discovery was published; (b) Immortal time arises from using post-eligibility information to define exposure; (c) Baseline was set as the day when the discovery was published and all scientists who had a diagnosis of dementia or died before age 75 years were excluded; (d) Immortal time arises from using post-exposure information to define eligibility.

Suppose baseline was set as the day when the discovery was published (Figure 3a). Figure 3b shows the bias with the presence of a competing risk. The arrow from D_1+_ to E_2_ means the scientists who were not Nobel Prize winners at time 1 have to remain free of dementia from time 1 to 2 to be classified as Nobel Prize winners at time 2. If they have dementia between time 1 and 2, their follow-up ends when dementia occurs, so E_2_ = E_1_ = 0. The arrow from CR_1+_ to E_2_ means the scientists who were not Nobel Prize winners at time 1 have to be alive from time 1 to 2 to be classified as Nobel Prize winners at time 2. These arrows create open paths between E_2_ and D_2+_ and between E_2_ and CR_2+_. As CR_2+_ precludes the occurrence of D_2+_, an additional open path between E_2_ and D_2+_ is generated.

#### When immortal time arises from using post-exposure information to define eligibility

Suppose baseline was set as the day when the discovery was published. All scientists who had a diagnosis of dementia or died before age 75 years were excluded (Figure 3c). Figure 3d shows the bias with the presence of a competing risk. The boxes around D_1+_ and CR_1+_ mean the analysis was restricted to scientists who remained alive without the occurrence of dementia until time 1+. Although these boxes close the open backdoor paths between E_2_ and D_2+_ and between E_2_ and CR_2+_, they create two open paths between E_1_ and U_1_. The exception is when the exposure, winning a Nobel Prize, has no causal effect on either the outcome, dementia, or the competing risk, cardiovascular death; then neither D_1+_ nor CR_1+_ is a collider (Supplementary Figure S4).

Competing risks should be particularly considered in studies investigating late-onset diseases among patients or older people. For example, a study investigating the effect of statin use on the risk of prostate cancer among patients with heart disease, has cardiovascular death as a competing risk that cannot be ignored. Competing risks should be considered more in studies involving older people, because disease rates usually increase with age.

### Solutions to address bias from immortal time

Table 1 summarizes the structures and sources of bias from immortal time. To avoid bias from immortal time, baseline should be the time when exposure allocation and eligibility for study inclusion are synchronized.^29^ Individuals with prior exposure should be excluded,^30^ because prior exposure may affect survival to recruitment and generate selection bias. All confounders and selection bias need to be adequately addressed to ensure exchangeability between groups at baseline, although this is not always feasible.^31^

A target trial framework helps align baseline, exposure allocation, and eligibility.^29^ An analogue of the intention-to-treat analysis sets baseline as the day when the discovery was published and defines Nobel Prize winners based on exposure status at baseline. This approach estimates the causal effect of E_1_ on D_2+_, which should not be confounded by D_1+_ that occurs after time 1 (Supplementary Figure S5). However, this approach is not practical here, because no scientist won a Nobel Prize when their discovery was published. Similarly, in a pharmacoepidemiological study where few individuals start treatment at eligibility, the analogue of the intention-to-treat effect estimates could be uninformative. In this case, the new-user design is more feasible.^32^ The new-user design identifies new users of the study drug and a comparator drug, matches the two groups based on when they start the drugs, and set baseline as the same time.^32^ This approach conditions on D_1+_= 0 because both groups have to remain alive until they start the drugs to be eligible; however, it does not introduce selection bias because selecting on E_1_= 0 (no one uses any drug before time 1+) closes the backdoor path between E_1_ and U_1_ generated by conditioning on D_1+_ (Supplementary Figure S6).

Alternatively, statistical approaches which focus on handling follow-up times correctly can be considered as an analogue of the per-protocol analysis that sets baseline at different eligibility times and considers each individual at each eligible time as different individuals. For example, the person-time approach accounts for person-time before winning the first Nobel Prize as unexposed and after that as exposed.^6^ Time-dependent analysis codes the exposure status as a time-varying variable that changes from 0 to 1 when scientists win their first Nobel Prize.^9^ The sequential approach emulates a sequence of mini trials with increasing baseline when eligible scientists who have not won a Nobel Prize before are classified into either group based on their exposure status within each mini trial; scientists in the unexposed group are artificially censored when they win their first Nobel Prize.^33^ The analogue of the per-protocol effect estimates are the combination of trial-specific estimates from each mini trial,^33^ that is the combination of the arrows from E_0_ to D_0+_, from E_1_ to D_1+_, … and from E_k_ to D_k+_ (Supplementary Figure S7). These approaches do not remove the arrows from D_0+_ to E_1_, from D_1+_ to E_2_, … and from D_k-1+_ to E_k_, because the scientists who were not Nobel Prize winners at time 0 still have to remain alive from time 0 to 1 to be classified as Nobel Prize winners at time 1, etc. Hence, differences persist between those who did and did not survive until the award, which requires addressing not only confounders and selection bias at baseline but also time-varying confounders that affect change in exposure status/artificial censoring and the outcome.^34^

## Conclusion

The fundamental cause of immortal time is misalignment of exposure allocation and eligibility. Specifically, immortal time arises from using post-eligibility information to define exposure or using post-exposure information to define eligibility. The structures of bias from immortal time are confounding by survival until exposure allocation or selection bias from selecting on survival until eligibility. Excluding immortal time from follow-up does not fully address the bias, and the presence of competing risks can worsen the bias. Epidemiological studies should be designed and analysed using rigorous approaches to avoid or mitigate bias from immortal time.

## Supplementary Material

Supplementary material

## Figures and Tables

**Table 1:** A summary of the structures and sources of bias from immortal time.

Cause of immortal time	Exclusion of immortal time	Presence of competing risks^a^	Structure and source of bias from immortal time	Directed acyclic graphs
Define exposure by post-eligibility information	No	No	Confounding by survival until exposure allocation (classical immortal time bias)	Figure 1b
Define eligibility by post-exposure information	No	No	Selection bias from selecting on survival until eligibility	Figure 1d
Define exposure by post-eligibility information	Yes	No	Confounding by survival until exposure allocation	Figure 2b
Define eligibility by post-exposure information	Yes	No	Selection bias from selecting on survival until eligibility	Figure 2d
Define exposure by post-eligibility information	No	Yes	Confounding by survival without occurrence of the outcome until exposure allocation	Figure 3b
Define eligibility by post-exposure information	No	Yes	Selection bias by selecting on survival without occurrence of the outcome until eligibility	Figure 3d

a There is no competing risk when the outcome is all-cause mortality; however, competing risks should be considered for all other outcomes.

## Data Availability

No new data were generated or analysed in support of this study.

## Key messages

- This study explains the fundamental causes of bias from immortal time, uses directed acyclic graphs to illustrate the structures of this bias, and further explores the structures of bias with the exclusion of immortal time and with the presence of competing risks.
- Immortal time arises from misalignment of exposure allocation and eligibility; the structures of bias from immortal time are confounding by survival until exposure allocation or selection bias from selecting on survival until eligibility.
- Excluding immortal time from follow-up does not fully address bias from immortal time, and the presence of competing risks can worsen the bias.
