# Supplementary material for: Illustrating the structures of bias from immortal time using directed acyclic graphs
Source: Int J Epidemiol. Author manuscript; Available in PMC 2025 Mar 28. (PMC11706530; doi:10.1093/ije/dyae176)
Supplement: Supplementary material [file EMS203507-supplement-Supplementary_material.docx]

**Supplementary figures**

**Illustrating the structures of bias from immortal time using directed acyclic graphs**


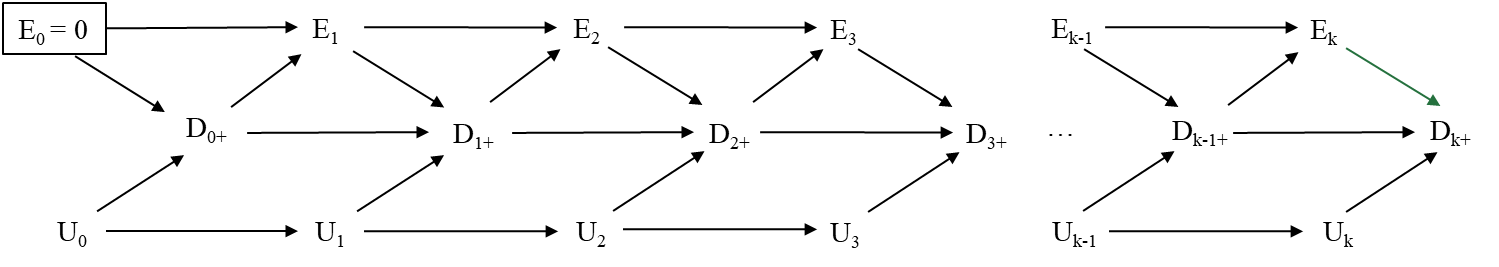


Supplementary Figure S1. Full directed acyclic graph from publication of the discovery (time 0) to the end of follow-up (time k+).

E_0,_ E_1_, … E_k_ are exposure status at time 0, time 1, … time k, respectively. D_0+_, D_1+_, …D_k+_ are outcome status between time 0 and 1, between time 1 and 2, …after time k, respectively. U_0_, U_1_, …U_k_ are status of another unmeasured cause of the outcome at time 0, time 1, … time k, respectively. Green arrow denotes the causal effect of interest, that is the effect of E_k_ (winning at least one Nobel Prize at or before time k) on D_k+_ (death at time k+). The box around E_0_ = 0 means no scientist won a Nobel Prize at or before time 0 when the discovery was published.


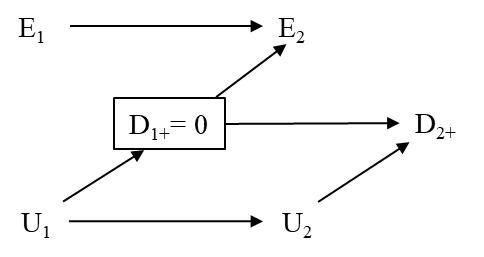

Supplementary Figure S2. Directed acyclic graph of the study design with immortal time, where immortal time arises from using post-exposure information to define eligibility and the exposure has no causal effect on the outcome.

E_1_ and E_2_ are exposure status at time 1 and 2, respectively. D_1+_ and D_2+_ are outcome status between time 1 and 2 and after time 2, respectively. U_1_ and U_2_ are status of another unmeasured cause of the outcome at time 1 and 2, respectively. The causal effect of interest is the effect of E_2_ on D_2+_.


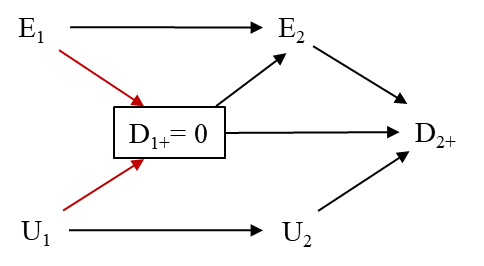


Supplementary Figure S3. Directed acyclic graph of the landmark approach.

E_1_ and E_2_ are exposure status at time 1 and 2, respectively. D_1+_ and D_2+_ are outcome status between time 1 and 2 and after time 2, respectively. U_1_ and U_2_ are status of another unmeasured cause of the outcome at time 1 and 2, respectively. The causal effect of interest is the effect of E_1_ on D_2+_. Red arrows denote key arrows that create open paths and result in bias.


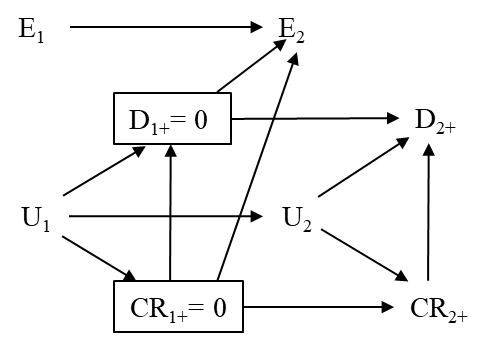


Supplementary Figure S4. Directed acyclic graph of the study design with immortal time and the presence of a competing risk, where immortal time arises from using post-exposure information to define eligibility and the exposure has no causal effect on the outcome or the competing risk.

E_1_ and E_2_ are exposure status at time 1 and 2, respectively. D_1+_ and D_2+_ are outcome status between time 1 and 2 and after time 2, respectively. CR_1+_ and CR_2+_ are status of a competing risk between time 1 and 2 and after time 2, respectively. U_1_ and U_2_ are status of an unmeasured common cause of the outcome and the competing risk at time 1 and 2, respectively. The causal effect of interest is the effect of E_2_ on D_2+_.


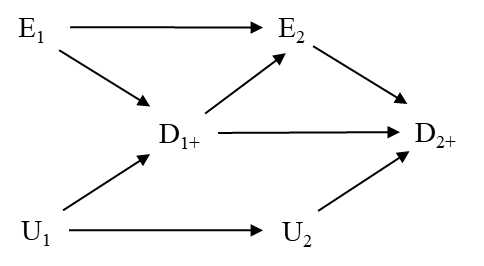


Supplementary Figure S5. Directed acyclic graph of an analogue of the intention-to-treat analysis.

E_1_ and E_2_ are exposure status at time 1 and 2, respectively. D_1+_ and D_2+_ are outcome status between time 1 and 2 and after time 2, respectively. U_1_ and U_2_ are status of another unmeasured cause of the outcome at time 1 and 2, respectively. The causal effect of interest is the effect of E_1_ on D_2+_.


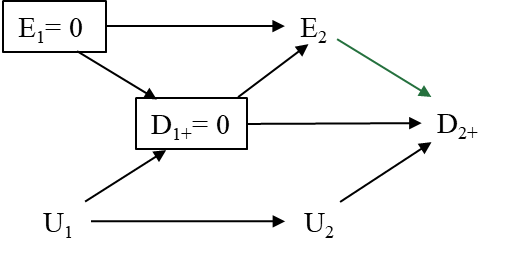


Supplementary Figure S6. Directed acyclic graph of the new-user design.

E_1_ and E_2_ are exposure status at time 1 and 2, respectively. D_1+_ and D_2+_ are outcome status between time 1 and 2 and after time 2, respectively. U_1_ and U_2_ are status of another unmeasured cause of the outcome at time 1 and 2, respectively. Green arrow denotes the causal effect of interest.


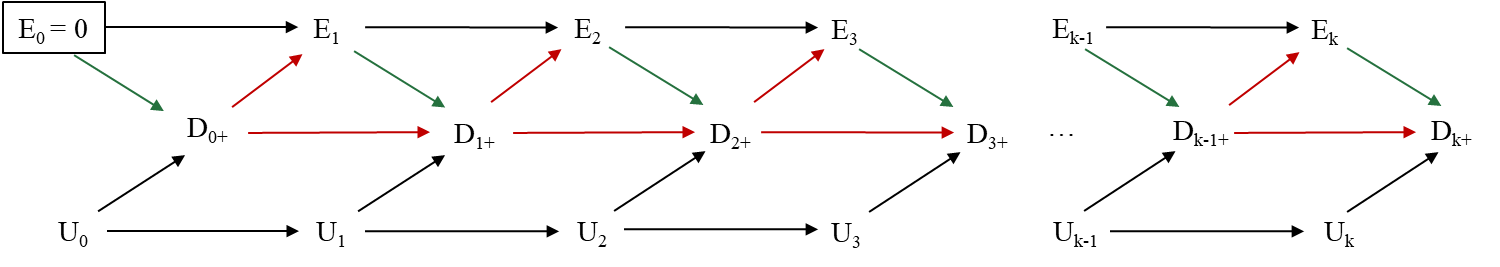


Supplementary Figure S7. Directed acyclic graph of an analogue of the per-protocol analysis that sets baseline at different eligibility times and considers each individual at each eligible time as different individuals.

E_0,_ E_1_, … E_k_ are exposure status at time 0, time 1, … time k, respectively. D_0+_, D_1+_, …D_k+_ are outcome status between time 0 and 1, between time 1 and 2, …after time k, respectively. U_0_, U_1_, …U_k_ are status of another unmeasured cause of the outcome at time 0, time 1, … time k, respectively. The causal effect of interest is the combination of all green arrows. Red arrows denote key arrows that create open paths and result in bias. The box around E_0_ = 0 means no scientist won a Nobel Prize at or before time 0 when the discovery was published.
